# A comprehensive review of mental health services across selected countries in sub-Saharan Africa: assessing progress, challenges, and future direction

**DOI:** 10.1007/s44192-025-00177-7

**Published:** 2025-04-07

**Authors:** Faith Atewologun, Olaniyi Abideen Adigun, Olalekan John Okesanya, Hakeem Kayode Hassan, Olaleke Noah Olabode, Abioye Sunday Micheal, Mohamed Mustaf Ahmed, Bonaventure Michael Ukoaka, Nimat Bola Idris, Tolutope Adebimpe Oso, Don Eliseo Lucero-Prisno

**Affiliations:** 1 Department of Medicine and Surgery, Ladoke Akintola University of Technology, Ogbomoso, Nigeria; 2 Department of Medical Laboratory Science, University College Hospital, Ibadan, Nigeria; 3 Department of Public Health and Maritime Transport, University of Thessaly, Volos, Greece; 4 Department of Medical Laboratory Science, Federal Medical Center, Bida, Nigeria; 5 Obafemi Awolowo University Teaching Hospital Complex, Ile-Ife, Nigeria; 6 Faculty of Basic Medical Sciences, Department of Public Health, Adeleke University, Ede, Nigeria; 7 Faculty of Medicine and Health Sciences, SIMAD University, Mogadishu, Somalia; 8 Department of Research and Innovations, Ehealth Somalia, Mogadishu, Somalia; 9 Department of Internal Medicine, Asokoro District Hospital, Abuja, Nigeria; 10 Department of Public Health, Al-Hikmah University, Ilorin, Nigeria; 11 Department of Medical Laboratory Science, Neuropsychiatric Hospital, Aro, Nigeria; 12 Department of Global Health and Development, London School of Hygiene and Tropical Medicine, London, UK; 13 Research and Development Office, Biliran Province State University, Naval, Philippines; 14 Research and Innovation Office, Southern Leyte State University, Sogod, Philippines

**Keywords:** Mental health services, Mental disorders, Primary health care, Rehabilitation, Community mental health services

## Abstract

Mental health is a crucial but frequently neglected aspect of general health and well-being that faces numerous challenges, including underfunding, shortage of trained professionals, pervasive stigma, inadequate infrastructure, and insufficient policies in sub-Saharan Africa. This review reports the significant progress and initiatives that have been made in this region. South Africa, Nigeria, Uganda, Kenya, Tanzania, Ethiopia, Rwanda, and Ghana have developed national policies and integrated mental health services into primary healthcare, marking a shift towards community-based care and reducing stigma through awareness campaigns. Countries such as South Africa and Rwanda have robust infrastructure, while Ethiopia and Kenya emphasize training primary healthcare providers and community-based models. Ghana’s Mental Health Act and Uganda’s collaboration with NGOs has enhanced awareness and resource mobilization. Mental healthcare-targeted programs, such as Kenya’s Friendship Bench and Ethiopia’s Health Extension Program, have demonstrated the efficacy of community-based interventions. South Africa has leveraged innovative approaches, such as telepsychiatry, to expand access to holistic mental health services, particularly in rural areas. Partnerships with traditional healers in Uganda and Rwanda have enhanced early identification and referral. Despite these advancements, challenges persist. Common issues include a severe shortage of mental health professionals, inadequate budget allocation, limited access to services in rural areas, and the need for comprehensive policy frameworks that continue to stall the desired goals. Urban-centric services in Nigeria, financial constraints in Tanzania, and resource limitations in Ethiopia and Rwanda have hindered equitable access. While public–private partnerships and technological innovations are emerging, the dominance of institutionalized care in several regions limits community outreach. To chart the path forward, improving mental health services in Sub-Saharan Africa requires increased funding, expanded training programs for mental health professionals, and incorporation of mental health into basic healthcare systems. Utilizing technology such as telemedicine and mobile health applications holds promise for overcoming geographical barriers and supporting ongoing education. Community-based models and advocacy efforts are essential for reducing stigma, promoting sustainable mental healthcare, and enhancing the overall well-being of citizens insub-Saharan Africa.

## Background

Mental health remains apivotal yet often suppressed component of overall health and well-being. In 2017, the World Health Organization estimated that approximately 450 million people suffered from mental illness globally [1]. Most communities in sub-Saharan Africa (SSA) grapple with the burden of mental health disorders, especially as less attention is given to mental health in the region. The fight against infectious diseases stretches the region’s health systems; mental health issues add another significant challenge [2]. Mental health issues in this region are pervasive and affect millions of individuals across diverse demographic groups [3].

**Fig. 1:**
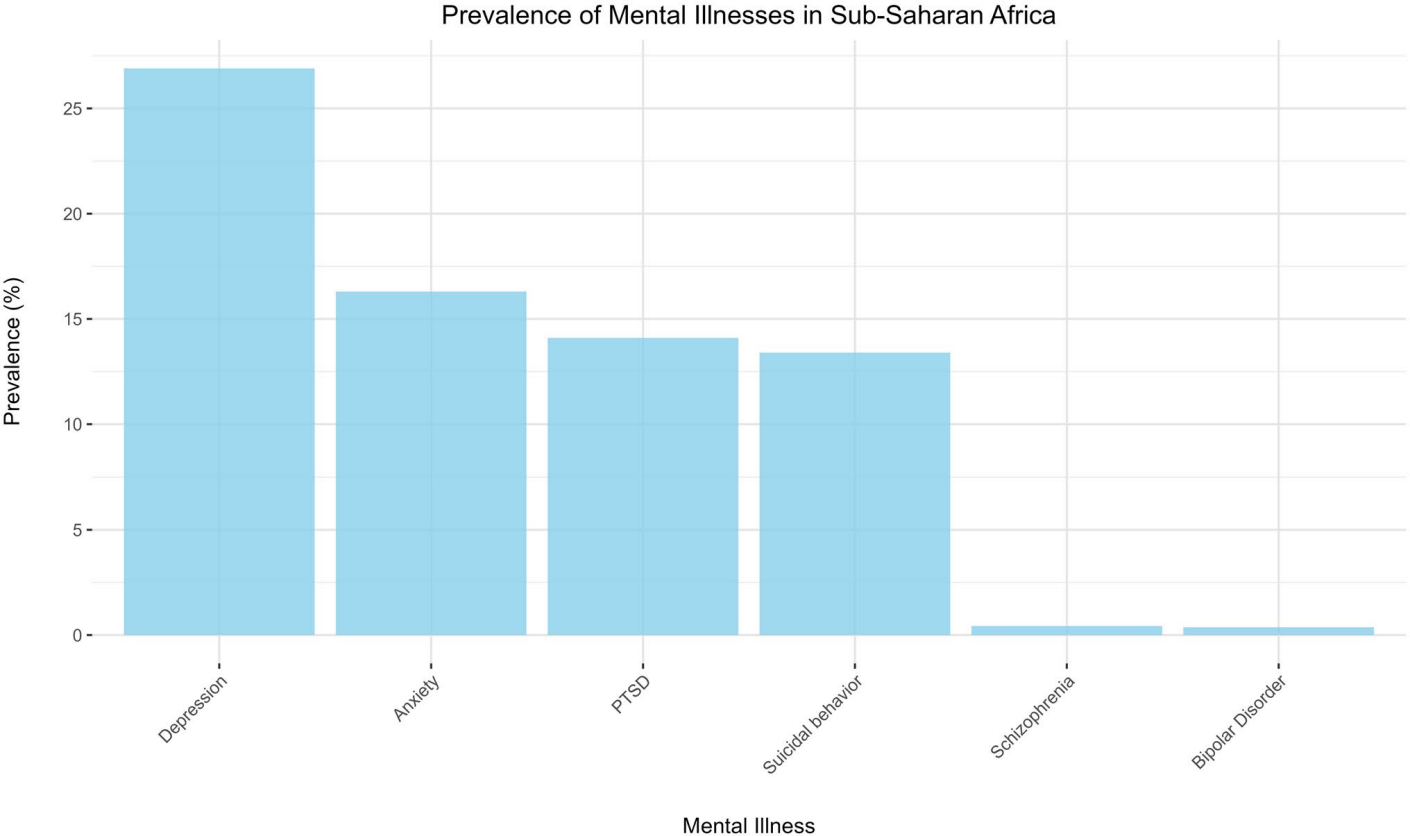
Prevalence of Mental Illnesses in Sub-Saharan Africa [3, 22]

The prevalence of mental illnesses, such as depression, anxiety, and schizophrenia, is substantial, and the burden is exacerbated by factors such as economic instability, conflict, and limited access to healthcare (Fig. 1). For instance, depression affects 26.9% of the population in SSA, which is markedly higher than the global prevalence range of 10–20% [4]. The mental health statistics across SSA reveal significant variations between countries. For example, Angola has a depression rate of 3.6 [5], Benin 11.6 [6], and Botswana and Burkina Faso show prevalence rates of 4.7 and 3.6, respectively [4, 7]. Nations like Kenya, Lesotho, and Liberia exhibit rates of 4.4, 4.8, and 3.5, respectively [4, 8, 9]. Meanwhile, countries such as Mauritius 4.4 and Rwanda 3.8 show notable mental health concerns [8], others like Niger 3.4 [4] and Guinea 3.9 [10] reflect more specific patterns. Other nations with reported ranges include Nigeria 3.9 [4], South Africa 4.6 [4], and Uganda 4.6 [11], underscoring the diversity in mental health challenges and needs across the region, with prevalence rates spanning a wide range of values. Similarly, anxiety disorders are reported by 16.3% of individuals in SSA compared to a global range of 10–30% [4].

Posttraumatic stress disorder (PTSD) has a prevalence of 22% among 10 of the 48 SSA countries in Africa, according to a systematic and meta-analysis conducted by Lauren et al., which is considerably higher than that in Europe (3.5%) and Asia (1.5%) [12]. Ward et al. found that the lifetime prevalence of PTSD among South African adolescents (aged 15–17) was 1.6%, based on a sample of 5631 individuals [13]. Shangani et al. found a striking 72.9% prevalence of PTSD in 655 Kenyan adolescents (mean age, 14 years) living in extreme poverty, with older adolescents showing higher rates of psychopathology [14]. In South Africa, Cortina et al. reported a 24.0% prevalence of posttraumatic stress symptoms among 1025 socially disadvantaged young adolescents (aged 10–12 years), with older age linked to higher rates [15]. In Burkina Faso, 17.8% of 360 adolescents (mean age 12.6) from ultra-poor villages exhibited PTSD symptoms, with violence exposure correlating with higher rates of depression and trauma [16]. A survey of 1025 students aged 10–12 years in rural South Africa revealed that social behavior disorders (15.2%) and behavioral and emotional disorders (41.0%) were more common than self-reported depression and anxiety (14.1%) [15], while 13.5% of behavioral and emotional disorders were reported in KwaZulu-Natal, South Africa [17]. The prevalence of suicidal behaviors among adolescents varies across populations. Suicidal behavior was also more prevalent in SSA (13.4%) than in Europe (9.2%) or Asia 4.8%) [3]. The 2008 South African Youth and Risk Behavior Survey (YRBS) revealed that 19.0% of students reported suicidal ideation and 21.8% had attempted suicide at least once in the past 6 months [18]. In Durban, Vawda et al. reported a 22.5% prevalence of suicidal ideation, 5.9% for suicide plans, and 5.4% for suicide attempts among 222 grade 8 learners [19]. Gage et al. reported a lower prevalence of suicidal ideation (11.2%) and attempts (2.3%) among 2, 079 adolescent girls in Ethiopia [20]. Zietz et al. found that 16.0% of adolescents in the HIV-endemic Nyanza region of Kenya reported suicidal ideation, with 12.0% considered to be at a high risk for suicide attempts [21]. The prevalence of schizophrenia in this region is 0.43%, which is slightly higher than the global prevalence of 0.32% [22]. In addition, stigma and cultural misconceptions regarding mental health often prevent individuals from seeking help [23]. Despite this critical need, mental health services in sub-Saharan Africa are typically underresourced and inadequately integrated into the broader health system. Although the current mental health professional proportion in Africa is not well estimated, 5 years data from the National Mental Health Strategy has shown a progressive rise in the number of psychiatrists [24]. A significant gap exists between the demand for mental health services and their availability, particularly in rural and underserved areas.

Mental health rehabilitation services are essential for aiding individuals with mental health conditions on their path to recovery, enabling them to reintegrate into society. These services encompass various interventions, including medical care, psychological counseling, social support, and vocational training, designed to help individuals regain their independence and enhance their overall quality of life [25]. This review delves into the current landscape of mental health services across selected countries in Sub-Saharan Africa. It highlights the progress made, identifies the ongoing challenges, and proposes future directions for improvement.

### Mental health services across selected countries in sub-saharan Africa

Mental health services in SSA vary greatly, with differences shaped by factors, such as healthcare infrastructure, government policies, and community involvement in mental health initiatives. This variation is evident in countries that have made significant strides in integrating mental health into their healthcare systems and in establishing community-based programs. Ethiopia, Nigeria, Rwanda, and Ghana were selected because of their progress in these areas. Ethiopia has developed community-based mental health initiatives, Nigeria has made efforts to integrate mental health services into primary healthcare, Rwanda has implemented national mental health strategies, and Ghana has incorporated mental health into the public health sector. Despite these advancements, resource limitations and stigma remain ongoing challenges across regions [1].

#### The current state of mental health services in Nigeria

Historically, Nigeria’s mental health services have largely focused on hospital-based care, primarily concentrated in urban areas, and often lack resources to meet growing demand. Federal neuropsychiatric hospitals, notably those in Yaba (Lagos) and Aro (Abeokuta), provide comprehensive psychiatric care, including inpatient and outpatient services, and manage severe cases of mental illness. They offer a range of rehabilitation services such as occupational therapy [26], which helps patients develop skills for daily living and employment, and social skills training, which focuses on improving interpersonal interactions and community integration. Despite the population exceeding 200 million, less than 10% of Nigerians have access to professional mental health services, even though 25–30% of the population experiences mental illness. With only 300 psychiatrists serving this vast population, there is a significant shortage of mental health professionals relative to the population size. Access to these services is further restricted because most of the population lives in rural areas, where mental health facilities are scarce. The concentration of psychiatric services in a few major cities creates a significant barrier to care for many Nigerians, especially those in underserved regions [27]. Moreover, only approximately three percent of the government’s health budget is allocated to mental health, underscoring the inadequate funding and low prioritization of mental health services by the government [27]. While challenges persist, there is a growing movement to decentralize mental health services and integrate them into primary care. This approach seeks to increase accessibility and reduce the stigma surrounding mental health treatments.

#### The current state of mental health services in South Africa

South Africa’s mental health infrastructure is relatively well developed compared to many other sub-Saharan African countries. The country’s mental health services are largely centered on hospitals, with specialized psychiatric hospitals and units within general hospitals providing a wide range of care. Cape Town’s Valkenberg Hospital and Weskoppies Hospital in Pretoria are key institutions in South Africa’s psychiatric care systems. These hospitals offer comprehensive inpatient and outpatient services including acute psychiatric care, long-term rehabilitation, and community reintegration programs. One of the key rehabilitation services provided in these hospitals is occupational therapy, which helps patients to develop the skills required for daily living and employment. Social skills training is a critical component that focuses on improving interpersonal interaction and community participation.

While South Africa has a relatively well-developed mental health infrastructure and robust national mental health policy framework, challenges persist in bridging the gap between the prevalence of mental illness and access to treatment. For instance, 16.5% of the adult populations suffer from mental illness, but only 25% receive treatment. This highlights significant barriers to accessing mental health services, including stigma and resource limitations [28]. Although the current proportion of mental health professionals is not well known, data from 5 years ago, as reported in the National Mental Health Strategy (2020–2025), indicate a significant increase in the number of psychiatrists [24]. South Africa has a well-established National Mental Health Policy Framework and Strategic Plan (2023–2030) [29] that aligns with the WHO-AIMS standards, disparities in access to mental health services remain a concern. A substantial proportion of mental health care (56%) occurs in institutionalized settings, emphasizing the need for greater investment in community-based services to improve accessibility and integration into daily life. There are 290 registered psychiatrists in the country, which is still insufficient for the population in need. This shortage was compounded by the uneven distribution of psychiatrists, particularly in rural areas. The South Africa Depression and Anxiety Group (SADAG) saw calls to its helpline double at the beginning of the country’s COVID- 19 lockdown, with an increase of 53% in the volume of calls from the previous year, receiving around 1400 calls per day. This indicates a surge in mental illness and demand for support exacerbated by the pandemic [30].

South Africa has made significant strides in implementing a more community-based approach to mental health care, transitioning away from institutionalized care. This shift is driven by policies such as the National Mental Health Policy Framework and Strategic Plan (2023–2030), which focuses on decentralizing mental health services, integrating them into primary healthcare settings, and developing accessible community-based services [29]. Mental health services in South Africa have seen significant growth in recent years, prioritizing community-based approaches. This shift emphasizes the importance of integrating mental healthcare into primary healthcare settings and developing accessible services closer to where people live. Outpatient clinics, mobile units, and home-based care programmes are examples of community-oriented initiatives. Such services aim to enhance the accessibility and continuity of care by providing mental health support in familiar environments. For instance, Western Cape Province has established a network of mental health centers in the neighborhood that offer a comprehensive range of services, from initial assessment to long-term rehabilitation and support. Programs such as District Mental Health Services provide comprehensive care including psychiatric consultation, psychotherapy, medication management, and support groups.

South Africa is exploring innovative approaches to enhance mental healthcare delivery, although some claims about the current telepsychiatry success rates require careful reevaluation. While telepsychiatry offers significant potential in an increasingly digitized environment, with over 90% of South African households having access to either a landline or mobile phone, its adoption remains aspirational rather than fully realized. Existing services primarily focus on remote consultations, follow-up care, and mental health education, leveraging mobile technology to bridge the gap in service provision [31]. ITWeb Census data reveals the fall of landlines and radios in South Africa, Johannesburg [30, 32]. Furthermore, South Africa has implemented several successful public–private partnerships to improve mental health care. For example, the Akeso Clinics Group offers a network of private psychiatric hospitals that provide high-quality inpatient and outpatient care, including specialized rehabilitation programs [33, 34]

#### The current state of mental health services in Kenya

The current state of mental health services in Kenya reflects the country’s progress but still grapples with critical gaps. In 2010, Kenya enacted a new constitution and introduced a devolved system of government that divided health responsibilities between national and county governments [35]. The national government manages health policy, referral facilities, and technical assistance, whereas county governments manage care delivery through local health facilities, pharmacies, ambulance services, and primary health care promotion. This localized approach aims to reduce disparities in mental healthcare access by tailoring services to county-specific needs [35]. However, challenges such as lack of county-specific policies, minimal budgetary allocation, insufficient human resources, limited integration into primary care, and inconsistent medication supplies limit the effectiveness of the devolved framework in achieving mental health equity. Inpatient and outpatient services are available, but remain centralized in referral hospitals [36]. Kenya has a national standalone mental health policy and legislative framework, in the form of the Mental Health Act of 1989. However, there are no county-specific mental health policies in Kenya, and counties still rely on outdated national legislation, despite having a mental health policy for (2015–2030) [37] and a National Mental Health Action Plan [38] aimed at integrating mental health care into primary care settings, decentralizing healthcare to county governments, and implementing capacity-building and community education programs. This policy demonstrates a substantial effort to improve mental health services and make them more accessible [36]. Additionally, only a few counties possessed physical copies of the national mental health policy, indicating the poor dissemination and accessibility of policy resources.

Recent data from the National Mental Health Strategy (2020–2025), spanning the period of 2013–2017, shows a significant increase in the number of psychiatrists from 60 to 68, indicating progress in the availability of mental health professionals. However, at the county level, there is little evidence of budgetary prioritization for mental health services, with only a few facilities allocated dedicated budgets for mental health care [24]. This financial gap underscores the systemic underfunding of mental health services, limiting the scope of the care provided. Inpatient mental health services are concentrated in a single purpose-built facility, the Moi Teaching and Referral Hospital, which has 80-bed capacity. Other countries, such as Trans Nzoia, Bungoma, and Busia, provide inpatient mental health care within general wards and lack specialized facilities. Involuntary admission practices, physical restraint, and seclusion were reported across all four facilities, reflecting outdated treatment approaches [36]. Outpatient mental health services are available in all four counties, with 12,220 patients treated during the fiscal year 2020/2021. Notably, 43.14% of these patients attended the national referral hospital, while 56.96% were treated at county referral facilities, illustrating a disparity in service utilization [36]. Psychosocial interventions, including psychotherapy, psychoeducation, counseling, social support, and rehabilitation activities, are available in these facilities and play a vital role in promoting recovery and reintegration for individuals with mental health disorders. Forensic mental health services are offered by three countries, providing outpatient treatment for offenders with mental illness. Kwobah et al. reported that human resource capacity for mental health services is critically insufficient, with only seven psychiatrists, 25 psychiatric nurses, 15 occupational therapists, 13 social workers, 18 psychologists, and 9 psychiatric clinical officers across three counties trained in mental health care, providing their services at high-level medical facilities with minimal structures at primary care facilities. This suggests that a limited number of mental health professionals are available at the primary care level, which can restrict access to care [36].

Kenya has taken significant steps in mental healthcare by developing community-based services. Integrating mental health into basic healthcare services is a central strategy supported by various initiatives and programs to provide accessible and culturally sensitive care, especially in suburban areas, where mental health services have historically been scarce [25, 39]. Despite the focus on community-based care, hospital-based services remain crucial for the treatment of severe mental health conditions. Institutions such as Mathari National Teaching and Referral Hospital in Nairobi provide specialized psychiatric care, including inpatient and outpatient services. For example, Mathari Hospital has established a rehabilitation unit that focuses on helping patients to reintegrate into their communities. The unit provides a range of mental health services to enhance the independence and quality of life of individuals with mental health disorders. Kenya has also embraced innovative approaches to enhance mental healthcare delivery. One such approach is the Basic Needs Basic Rights model [40], which integrates mental healthcare with livelihood support. This program provides mental health services, along with vocational training and income-generating activities, helping individuals with mental health conditions achieve economic independence. The Basic Needs Basic Rights model has been successfully implemented in several regions, improving both the economic stability and mental health outcomes of participants [40].

#### The current state of mental health services in Uganda

Uganda has made significant efforts toward mental health policy development and the development of community-based mental health care in sub-Saharan Africa. Mental health services in Uganda face significant challenges, including high prevalence rates, social stigma, and lack of resources [41]. An estimated 35% of Ugandans suffer from mental illnesses, with 15% requiring treatment; however, the actual figures may be much higher because of underreporting and limited research [42]. A study in Eastern Uganda revealed that 60.2% of respondents had a diagnosable mental illness, with the majority classified as moderate or severe [43]. Despite the high burden, approximately 90% of those affected never seek treatment according to the World Health Organization [42]. As one of the few African countries with a national mental health policy since 1996, Uganda’s policy aims to decentralize and integrate mental health services into primary health care (PHC), promote evidence-based treatments, involve families and communities, protect the rights of individuals with mental illness, and combat stigma [44–46]. For example, Mental Health Uganda (MHU), a prominent organization in this field, operates numerous community support groups and peer support programs across the country [44]. These groups provide a platform for individuals with mental health conditions to share experiences, receive support, and engage in therapeutic activities. Community health workers in Uganda are trained to identify and manage prevalent mental health conditions, which has contributed to improved access to mental health services in some regions. These workers conduct home visits, offer counseling, and facilitate referrals to specialized care when necessary. However, the integration of mental healthcare plans into the community health system remains uneven, with significant gaps in implementation across various districts. While these efforts have reduced the need for people to travel long distances to access care, challenges such as stigma and resource constraints persist [44].

Uganda faces a critical shortage of mental health professionals, with a rate of only 0.08 psychiatrists per 100,000 people, most concentrated in urban areas, creating disparities in care access between urban and rural regions [41, 45]. This statistic places Uganda among the countries with the lowest psychiatrist-to-population ratios worldwide, presenting a substantial barrier to professional mental healthcare access. Efforts to address this shortage through the training of psychiatric clinical officers and other mental health personnel are ongoing but insufficient to meet the growing demand [41]. Resource constraints are a major issue, with only 1% of the healthcare budget being allocated to mental health. Most funding focuses on treating severe psychiatric disorders in inpatient facilities, leaving common conditions like depression, anxiety, PTSD, and relational issues largely unaddressed [44, 46, 47].

Hospital-based mental health interventions in Uganda are primarily provided by regional referral hospitals and specialized psychiatric units. The Butabika National Referral Mental Hospital in Kampala is the largest psychiatric facility in the country offering comprehensive inpatient and outpatient services [30]. Butabika Hospital provides a range of rehabilitation services, including occupational therapy, vocational training, and social skill development. These services are designed to help patients recover and integrate into their communities. Despite its critical role, Butabika Hospital and other facilities face resource limitations including inadequate staffing, funding, and infrastructure [48]. Other regional hospitals, such as Gulu Regional Referral Hospital, also offer mental health services, although these are often limited by resource constraints. These hospitals contribute significantly to the management of severe mental health conditions that require intensive treatment and rehabilitation [49].

Cultural beliefs and misconceptions such as attributing mental illness to spirits and witchcraft further hinder access to evidence-based care. Many patients initially consult traditional healers who, although compassionate, often employ unscientific and harmful practices such as chaining patients. This delays medical intervention and reduces the likelihood of recovery [41]. Additionally, Molodynski et al. reported that 80% of the patients in mental hospitals sought traditional healers before accessing medical care [41, 42]. Furthermore, Uganda pioneered the integration of traditional healing interventions with modern mental healthcare. Traditional healers are often the first point of call for individuals with mental illness, particularly in rural areas. Recognizing this, the Health Ministry initiated collaborations with traditional healers to provide culturally sensitive care and encourage referrals to biomedical services when necessary [44, 50, 51]. Uganda has made significant strides in expanding access to mental health services through community-based initiatives and the inclusion of mental health services in primary healthcare [17].

YouBelong Uganda (YBU) established the YouBelong Home (YBH) intervention in Uganda to bridge the gap between hospital-based and community-based mental health care. The YBH model involves a pre-discharge phase and post-discharge empowerment plan that addresses stigma, poverty, and limited healthcare infrastructure, aligning with global recommendations for strengthening non-hospital-based mental health care [52, 53]. It incorporates WHO’s mhGAP Intervention Guide (mhGAP-IG) principles and collaborates with traditional healers. The intervention demonstrates the potential of holistic, community-centered mental health care models in Uganda, but systemic challenges such as resource limitations and a robust referral network highlight the need for innovative strategies [54]. The success of peer support programs and involvement of local health authorities have improved service delivery and reduced stigma. The innovative use of mobile phones has expanded the reach of mental health services to the underserved population.

#### The current state of mental health services in Ethiopia

Mental health disorders in Ethiopia affect 18% of adults and 15% of children, with significant needs across all age groups from early childhood through adolescence [55]. Ethiopia has taken a bold step in integrating mental health services into its primary healthcare system, guided by the National Mental Health Strategy, from 2012/13 to 2015/16. Ethiopia’s first National Mental Health Symposium, held in Addis Ababa on 11–12 August 2014, evaluated the country’s mental health challenges and explored effective strategies for their improvement. The event brought together mental health professionals, religious leaders, international organizations, and experts to discuss the reform and scale-up of mental health services in Ethiopia. The Health Extension Programme was highlighted as a key component enabling health extension workers to provide basic mental health support. The symposium underscored Ethiopia’s commitment to improving mental health services, reducing stigma, and integrating mental healthcare into the broader health system to address the needs of its population [56].

In collaboration with various international organizations, the Ethiopian government has developed programs to train primary healthcare workers in mental healthcare. These programs aim to decentralize mental health interventions, making them more open to rural dwellers, who constitute the majority of the country [31]. Primary mental health care providers in Ethiopia include health extension workers (HEWs), psychiatric nurses, and mid-level mental health professionals, such as those with a master’s degree in clinical and community mental health. HEWs play a critical role in community awareness, case identification, and referral to health centers or primary hospitals while also monitoring treatment adherence. These referrals are not made directly to tertiary hospitals, but rather follow a stepwise progression within the health system [57, 58]. At the health center and primary hospital levels, psychiatric nurses and mid-level mental health professionals diagnose, treat, and follow up with patients in outpatient departments, while stabilizing emergency psychiatric cases. In general hospitals, master-level clinical and community mental health professionals, along with psychiatric nurses, manage both outpatient and inpatient psychiatric services. At the tertiary hospital level, psychiatrists and consultants deliver specialized outpatient and inpatient care and accept referrals from primary healthcare settings and general hospitals [59–62].

Community Health Extension workers (CHEWs) play a crucial role in delivering mental health services at a community level. They were trained to identify common mental health disorders, provide basic counselling, and refer severe cases to tertiary health facilities [63, 64]. The incorporation of mental health services into the National Health Extension Program has improved early diagnosis and intervention in mental illness. Hospital-based mental health services in Ethiopia are provided by a few specialized psychiatric hospitals and psychiatric units in general hospitals [57, 65, 66].

The Amanuel Mental Specialized Hospital in Addis Ababa is the primary psychiatric facility in the country, offering numerous services including inpatient care, outpatient care, and rehabilitation programs. The hospital provides comprehensive rehabilitation services such as occupational therapy, which helps patients develop skills for daily living and employment, and psychosocial rehabilitation, which focuses on improving social functioning and community integration [67]. However, 15% of Ethiopians are affected by major mental illnesses or substance abuse disorders, and a population of over 100 million is left to be cared for by approximately 60 psychiatrists [68, 69]. This highlights a severe shortage of psychiatrists relative to the population size. Although the current mental health professional proportion in Ethiopia is not well known, the National Mental Health Strategy, 2020–2025 (2013–2017 EFY) reveals a significant rise in the figure [24]. Nevertheless, psychiatric care is being delivered across all levels of the health system by a range of trained professionals, including HEWs, psychiatric nurses, and mid-level mental health professionals, which expands access to care beyond tertiary hospitals in Addis Ababa [31, 58, 70].

Other general hospitals in major cities have psychiatric units offering basic mental health services. However, these services are not readily available because there is a shortage of mental health specialists within the country. Despite these challenges, 3500 people received mental health care as part of the WHO Mental Health Gap Action Programme, showing that there are efforts and programs in place to provide mental health care to the population, even if their reach is currently limited [31]. Ethiopia has embraced innovative approaches to enhance the delivery of mental healthcare. One notable initiative is the Mental Health Gap Action Programme (mhGAP) [71], developed by the World Health Organization (WHO), which aims to scale up services for mental illnesses, neurological diseases, and substance-use disorders. The mhGAP-Intervention Guide (mhGAP-IG) has been implemented in several regions to train primary healthcare providers to identify and manage mental health disorders [72]. However, cultural beliefs often shape how mental health is understood and addressed. For instance, many Ethiopian mothers with perinatal depressive symptoms tend to attribute their distress to external factors, such as poverty or supernatural causes, rather than recognizing it as mental health-related. Similarly, Ethiopian parents are often hesitant to collaborate with non-traditional professionals, even if they acknowledge their children’s need for treatment because of cultural beliefs [55].

#### The current state of mental health services in Tanzania

The first-ever National Mental Health Dialogue in Dar es Salaam, held in commemoration of World Mental Health Day 2022, emphasized the critical importance of prioritizing mental health. The event, themed “Make Mental Health and Wellbeing for All a Global Priority,” gathered key stakeholders, including the Minister for Health and other prominent officials, alongside representatives from WHO and UNICEF. The report also stressed that stigma, misinformation, and inadequate services contributed to the burden of mental health conditions, with individuals suffering from mental illness potentially losing 10–20 years of life [73]. Tanzania has initiated efforts to incorporate mental health services into its primary health scheme, although progress has been slower than in other countries in the region [74]. The Health Ministry, along with the Ministry of Social Development, Gender, Elderly, and Children, has issued a guide for incorporating mental health into primary care, emphasizing the training of primary healthcare providers and the development of community-based mental health programs. Community Health Volunteers (CHVs) are trained to identify common mental disorders, provide basic counseling, and facilitate referrals to specialized services. These workers conduct home visits and community outreach programs, increasing their knowledge of mental illness and reducing stigma. Hospital-based mental health services in Tanzania are provided by top-tier psychiatric hospitals, and psychiatric units in general hospitals. Muhimbili National Hospital, at the heart of Dar es Salaam, houses the country’s primary psychiatric facility and offers comprehensive inpatient and outpatient services. The hospital provides a range of rehabilitation services, including occupational therapy, vocational training, and social skill development. Other regional and district hospitals also offer mental health services, although they are often limited by resource constraints. These hospitals play a crucial role in managing severe mental health conditions that require intensive treatment and rehabilitation [75–77].

In a cross-sectional study of the psychological state of people between April and October 2019 in the Mbeya and Songwe regions of Tanzania, 78.4% of participants reported that they were likely to be well, while 13.4% reported mild, 5.7% moderate, and 2.6% severe psychological distress [76]. This indicates that a significant proportion of the population experiences varying levels of psychological distress, highlighting the need for effective mental health interventions. Tanzania has implemented several creative methods to improve mental health care delivery. One such initiative is the inclusion of mental health screening services in routine health checkups at primary health care centers. This approach helps identify mental health issues early and provides an opportunity for timely intervention [78]. A study examining mental health services in Tanzania revealed that many patients lack adequate support, particularly in social participation and self-care, as caregivers and community members often lack awareness of how to effectively assist individuals with mental disorders. The limited involvement of patients in home and community activities is a key issue, often due to misconceptions about their capabilities or the side effects of psychotropic medications. Self-care deficits and emotional support are significant barriers to recovery, and caregivers play a crucial role. Financial constraints limit access to medications and follow-up care, particularly for patients with severe mental illness [79]. Another study by Ambikile et al. [77] revealed significant challenges in improving home care and support for individuals with mental illness in Tanzania, stating key issues, such as poor understanding of mental illness among caregivers, abandonment of care responsibilities, disputes over treatment modalities, and lack of outreach mental health services. Ambikile et al. also reported that caregivers often lack awareness of mental illnesses, leading to cultural misconceptions and stigma. The lack of outreach mental health services is a critical gap that requires strategic resource allocation and policy support. Community engagement, strengthened policy frameworks, and collaboration between traditional and formal health systems are essential for closing the treatment gap in Tanzania [80].

In Tanzania, digital mental health platforms were most commonly used for assessments (40.9%), followed by teletherapy (24.6%), telecoaching (21.7%), and a combination of both services (12.9%). Those who used telecoaching only completed an average of 3.2 sessions, while those who relied solely on teletherapy completed an average of five sessions. Of those who used both services, the majority (73.1%) began telecoaching, 25.6% started teletherapy, and 1.3% initiated both services on the same day. Participants who utilized telecoaching, teletherapy, or a combination of both were more likely to show clinical improvement than those who only underwent assessment [81]. Additionally, Tanzania has adopted digital platforms to enhance mental healthcare. The Mental Health and Psychosocial Support (MHPSS) program uses mobile technology to provide mental health education, self-help tools, and tele-counseling services [82]. This approach has been particularly effective in reaching young people and individuals in remote areas [83].

#### The current state of mental health services in Rwanda

Rwanda made significant strides in developing community-based mental health services, particularly following the 1994 genocide [84]. With the support of various international organizations, the government has implemented a range of interventions to address the mental health requirements of its citizens. The Mental Health Policy and Strategic Plan outlines the inclusion of mental health services in primary health care and the establishment of local support systems. Institutional-based mental health services in Rwanda are provided by the Ndera Neuropsychiatric Hospital in Kigali and by psychiatric units within regional hospitals. Ndera Hospital offers comprehensive inpatient and outpatient services including acute psychiatric care, rehabilitation, and community reintegration programs. Mental health rehabilitation services at Ndera Hospital focus on occupational therapy, vocational training, and social skills development to help patients reintegrate into their communities [85]. Regional hospitals also provide mental health services although they are often limited by resource constraints. These hospitals play a crucial role in managing severe mental health conditions and providing follow-up care after discharge from specialized facilities, such as Ndera Hospital.

Rwanda implemented several innovative approaches to improve mental health care delivery. One notable initiative is the inclusion of mental health services in the community-based health insurance program Mutuelle de Santé, which covers over 90% of Rwanda’s population, including mental health services in its package [86]. This integration increases mental health service utilization for the public and reduces financial barriers to care [87]. Additionally, Rwanda embraces the use of technology to enhance mental healthcare. In collaboration with various partners, the Health Ministry of Health has developed digital platforms to provide mental health education, self-help tools, and tele-counselling services. Rwanda has implemented digital mental health initiatives, such as the “Babyl” system, which provides virtual consultations leading to increased access to healthcare services, especially for rural populations where high user satisfaction was recorded. This service has had over two million active users and more than 1.3 million virtual services rendered since its launch in 2016 [86].

#### The current state of mental health rehabilitation services in Ghana

Ghana developed a strong framework for community-based mental health services guided by the National Mental Health Act of 2012 [88]. This legislation promotes the inclusion of mental health care into primary health care and the establishment of community-based mental health services. The Mental Health Authority (MHA) presides over the implementation of these services and ensures that mental healthcare is accessible to all. The MHA has developed a mental health information scheme that collects data on mental health interventions and outcomes, helping inform policies and improve service delivery. This system also includes a mobile application that provides mental health information and resources to the public [89]. A key element of Ghana’s community-based mental health service is the involvement of community psychiatric nurses. These nurses are trained to deliver mental health care directly within communities, conduct home visits, offer counseling, and facilitate referrals to specialized services. Additionally, community support groups and peer networks are essential in providing psychosocial support and helping reduce the isolation of individuals with mental health conditions. In 2011, there were 1068 registered mental-health nurses and 72 community mental-health officers in Ghana. Despite an estimated 2.4 million persons experiencing mental illness that year, only 2.8% received treatment, with most of these individuals located in the capital city. As a result, many others turned to informal healthcare providers, such as traditional healers and faith-based organizations, for help [90].

Mental health services in Ghana are underdeveloped, with less than 1% of individuals accessing necessary care [91]. According to the WHO Mental Health Reports in 2020, Ghana has three mental hospitals with 1171 beds, including two in the capital, Accra, and one in the Central region, offering outpatient services and drug rehabilitation units with plans to build more. The Accra Psychiatric Hospital has a dedicated ward for children and adolescents, housing 15 beds [92]. Hospital-based mental healthcare in Ghana is provided by specialist hospitals and psychiatric units in general hospitals. Psychiatric hospitals in Accra, Pantang Specialist Hospital, and Ankaful Psychiatric Hospital are the main psychiatric facilities in the country [93]. The primary conditions included substance use-related mental disorders (42%), schizophrenia (34%), and mood disorders (16%). In 2020, 1928 patients were treated, with 30% female and 1% children or adolescents. The average stay was 105 days, with 66% remaining for less than 1 year [92]. The rehabilitation services provided in these hospitals focused on helping patients develop skills for independent living and community reintegration. Occupational therapy, vocational training, and social skills development are the key components of these programs. Hospitals also work closely with community mental health services to ensure continuity of care for patients after discharge [94]. According to Weobong et al. [91], the key challenges in integrating district mental healthcare plans in Ghana include insufficient staffing, weak supervision systems, unreliable psychotropic medication supplies, and a lack of psychological treatments due to the absence of trained clinical psychologists. The health system also lacks functional mental healthcare plans, adequate human resources, and limited supervision and training [91]. Despite this, there are promising community support systems, such as volunteer networks and collaborations with traditional and faith-based providers. The Mental Health Act of 2012, a best-practice model, has not been fully implemented, hindering the integration of mental health services into primary health care [91]. Mobile health services, such as SMS-based support and mobile apps, have been promising for improving mental health outcomes in Ghana. For example, the MindME App provides immediate help to users during psychological distress episodes. The app uses Google Maps to locate and automatically place a distress call to the community mental health nurse closest to the user [91, 95]

### Assessing progress

Significant strides have been made in sub-Saharan Africa regarding mental health rehabilitation services, marked by notable policy advancements, successful programs, and increased awareness and advocacy (see Table 1). In recent years, various countries have developed and implemented national mental health policies and plans, reflecting growing awareness of the importance of mental wellness at the government level. For instance, Nigeria’s Mental Health Bill provides a comprehensive framework for mental health care encompassing funding, healthcare delivery, and patient rights protection [95]. Similarly, Uganda’s National Mental Health Policy focuses on incorporating mental health services into community-based services and primary health care. These significant policy advancements highlight governments’ commitment to systematically and sustainably address mental health issues to support community-based mental health care, which emphasizes de-institutionalization and reintegration of patients into society [96]. Uganda has also established a collaborative effort with NGOs to improve public awareness and mobilize resources for mental health services. These collaborations have facilitated community reintegration programs that focus on reducing the stigma associated with mental illness [97]. In addition to policy development, several successful programmes and interventions across the region have demonstrated significant progress in mental health services.

**Table 1:** Mental health services across selected countries in Sub-Saharan Africa

Country	Key progress	Key challenges
Ethiopia	Integration of mental health services into the primary healthcare system (National Mental Health Strategy)—Health Extension Program to support mental health at the community level—Mental Health Gap Action Programme (mhGAP) to train primary healthcare providers—Development of a national mental health symposium to discuss mental health reform—Community-based care through HEWs and mental health professionals	Severe shortage of psychiatrists (60 psychiatrists for a population of over 100 million)—Limited access to mental health infrastructure and specialized services in rural areas. Cultural misconceptions about mental health. Stigma and lack of awareness about mental illness
Nigeria	Federal neuropsychiatric hospitals (e.g., Yaba, Aro) provide comprehensive care and rehabilitation services like occupational therapy and social skills training—Growing movement to decentralize services into primary care to improve accessibility. Community-based rehabilitation services and awareness campaigns by the Asido Foundation	Severe shortage of mental health professionals (300 psychiatrists for a 200 + million population)—Only 3% of the health budget is allocated to mental health—Services concentrated in urban areas, leaving rural populations underserved, and inconsistent policy implementation
Rwanda	Integration of mental health into primary healthcare. Comprehensive mental health services at Ndera Neuropsychiatric Hospital. Inclusion of mental health services in the community-based health insurance program (Mutuelle de Santé). Use of technology for virtual consultations and mental health education. Support for community reintegration programs	Resource constraints in regional hospitals—Limited access to specialized mental health services in remote areas—Need for continued community engagement and mental health awareness
Ghana	Strong framework for community-based mental health services through the National Mental Health Act of 2012. Mental Health Authority ensures accessible mental healthcare—Development of a mental health information scheme. Increased public awareness of mental health. Involvement of community psychiatric nurses and use of digital platforms	Limited mental health workforce and resources—High stigma surrounding mental health—Inadequate mental health services in some regions—Barriers to accessing care in rural areas
South Africa	Well-developed infrastructure with specialized psychiatric hospitals like Valkenberg and Weskoppies. The National Mental Health Policy Framework (2023–2030) prioritizes decentralization and community-based care. Public–private partnerships, e.g., Akeso Clinics. Use of telepsychiatry to extend the reach of mental health services	High demand for services and uneven distribution of psychiatrists, particularly in rural areas—Only 25% of individuals with mental illnesses receive treatment—Institutionalized care still dominates, limiting the reach of community-based services
Tanzania	Incorporation of mental health services into primary care—Training of Community Health Volunteers (CHVs) to identify and provide basic care—Use of digital platforms for teletherapy and telecoaching—Mental health dialogue and national focus on mental health—Hospital-based mental health services provided by regional hospitals	Limited mental health professionals and cultural stigma, misinformation, and lack of awareness. Resource constraints in regional hospitals. Financial barriers to accessing medication and follow-up care. Limited support for home-based care. Lack of outreach services
Kenya	The devolved health system divides responsibilities between national and county governments—Community-based models like ‘Basic Needs Basic Rights’ integrate mental health with livelihood support—Outpatient and psychosocial services are available	Limited mental health professionals (e.g., 7 psychiatrists in select counties)—Centralized services, with inpatient care mostly at referral hospitals—Lack of county-specific policies and minimal budget allocation for mental health
Uganda	Incorporation of mental health services into primary health care; development of community-based mental health services. Significant policy development to support community-based mental health care—Programs addressing stigma and improving community reintegration of patients—Collaboration with NGOs for awareness and resource mobilization	High prevalence of mental illness with insufficient mental health professionals and facilities. Persistent social stigma and cultural barriers. Limited budgetary support and poor integration into primary healthcare systems

In Ethiopia, the integration of mental health services into the primary health care system through the National Mental Health Strategy is a notable achievement. This strategy seeks to make mental health services a routine part of healthcare delivery [96]. The Health Extension Program plays a crucial role in supporting mental health at the community level, ensuring that services reach the most vulnerable population. The Mental Health Gap Action Programme (mhGAP) in Ethiopia trains primary healthcare providers to better address mental health issues, expand the workforce, and improve service delivery [98]. Kenya’s Friendship Bench program is a Zimbabwean innovation pioneered in Zimbabwe, which utilizes problem-solving therapy through community health workers at the primary care level and has effectively masked the symptoms of anxiety and depression, showcasing the potential for scalable and sustainable mental health interventions. These programmatic successes highlight the impact of well-designed and implemented mental health initiatives [99]. Furthermore, considerable progress has been made in efforts to increase knowledge and reduce stereotypes associated with mental illness. Campaigns such as South Africa’s “Mental Health Awareness Month” and Nigeria’s “Mental Health Awareness Day” aim to educate the public and promote understanding of mental health issues [100, 101]. Additionally, various NGOs and advocacy groups have been instrumental in driving these initiatives and advocating better mental health policies and services [102, 103]. Increased media coverage and social media campaigns have further contributed to changing perceptions and encouraging individuals to seek help [104–106].

The advancements in policy, successful program implementation, and heightened awareness and advocacy efforts collectively signify substantial progress in mental health services in sub-Saharan Africa. These achievements provide a solid foundation for further improvements and highlight the potential for continued advancements in mental health care across the region. South Africa has achieved significant progress in mental health care through its comprehensive National Mental Health Policy Framework (2023–2030) and the development of hospital-based, decentralized care and expanded community-based mental health services, which is a significant advancement in addressing mental health needs at the grassroots level [107]. The inclusion of mental health in primary healthcare has improved accessibility and continuity of care, while innovative approaches such as telepsychiatry have extended the reach of mental health services to underserved areas [108]. Kenya’s health system has contributed to a huge leap in expanding its mental health services, particularly through the inclusion of mental healthcare into primary health systems, the development of community-based services, and the distribution of responsibilities for mental health between national and county governments, improving localized decision-making. Innovative programs such as the Basic-Needs model have demonstrated the potential of combining mental health care with livelihood support to enhance overall well-being [40].

Tanzania has made notable progress in expanding access to mental health services through the inclusion of mental healthcare in primary healthcare systems and the implementation of community-based programs. The involvement of community health volunteers and the use of digital platforms have improved service delivery and accessibility [83]. Ethiopia has made significant progress in expanding access to mental health care through the integration of mental health care into primary health systems and the implementation of community-based programs. The success of the Health Extension Program and mhGAP initiative has improved service delivery and reduced treatment inequalities for mental health conditions [99]. Rwanda has made significant progress by prioritizing the integration of mental health into primary healthcare, ensuring that mental health is addressed at all levels of care, from community health workers to specialist services [106]. Rwanda has also made mental health services available through community-based health insurance programs, ensuring that financial barriers are reduced for patients seeking care [109]. Ghana has made significant progress in expanding access to mental health services through the implementation of the National Mental Health Act, which provides a strong legislative framework that ensures that mental health services are accessible to the population and that community-based services are deployed [89]. The Mental Health Authority has been instrumental in organizing mental health services and providing access to care across the country. Increased public awareness campaigns have helped reduce stigma and foster a more supportive environment for people with mental health disorders [110].

### Challenges of mental health services in sub-Saharan Africa

Mental health services in sub-Saharan Africa face a myriad of challenges that hinder their effectiveness and accessibility. These challenges are multifaceted and encompass financial, structural, cultural, and political dimensions [111, 112]. One of the most pressing challenges is chronic underfunding of mental health services. Health budgets in many sub-Saharan African countries allocate a minimal percentage to mental health, often lower than one percent of the total health budget [112]. This lack of funding results in inadequate facilities, insufficient medications, and scarcity of well-trained mental health specialists [113]. For example, many countries have only one psychiatrist per million people, far below the recommended standard. The region also faces a severe shortage of mental health professionals including psychiatrists, clinical psychologists, psychiatric nurses, and social care workers [114]. Training programs for these professionals are limited, and often do not have adequate resources and support. In addition, a few trained professionals frequently migrate to countries with better working conditions and pay, exacerbating this shortage. Stigmatization and discrimination against individuals with mental illness are pervasive in sub-Saharan Africa [115]. Cultural beliefs and misconceptions regarding mental illness often lead to social exclusion and discrimination. Many communities perceive mental illness as an offshoot of a supernatural force or as a personal failure that discourages individuals from seeking help and adhering to treatment.

The infrastructure for mental health services is often underdeveloped, as many countries have few specialized psychiatric hospitals and those that exist are frequently located in urban areas, making them inaccessible to the rural population, which constitutes a significant portion of sub-Saharan Africa’s population [116]. Community-based services are limited, and there is a lack of mental health inclusion in primary health care systems. Moreover, mental health care in many sub-Saharan African countries remains hospital-centric with limited development of community-based rehabilitation services. For it to be effective, mental health services often require community support, including social reintegration programs and support groups that are sparse in the region [117]. The absence of these services has hampered the reintegration of individuals with mental illnesses into society.

Although some countries have developed mental health policies, many lack comprehensive legislative frameworks to respect the rights of individuals with mental health conditions and to ensure the provision of optimal services. The absence of such frameworks leads to inconsistencies in service delivery and the protection of the rights of patients with mental health problems. Public awareness of mental disorders remains low in many SSA countries [55]. There is a general lack of understanding of mental health conditions and available treatments, which contribute to stigma and delays in seeking care. Public awareness campaigns are not widespread, and, where they exist, they are often underfunded and limited in scope. Political instability and conflict in some SSA countries exacerbates the challenges experienced by mental health services. Conflicts lead to displacement, trauma, and increased mental health needs, while simultaneously disrupting health services and infrastructure [118]. The challenges experienced by mental health services in SSA are profound and multi-faceted. Addressing these issues requires concerted efforts from governments, international organizations, and local communities. Increased funding, periodic training, assessments, retention of mental healthcare professionals, enhanced infrastructure, reduced stigma, and incorporation of mental healthcare services into primary care are essential steps toward improving mental healthcare in the region [119]. Public awareness campaigns and policies that protect the rights of persons with mental disorders are crucial for overcoming these challenges. Despite these advancements, Ethiopia faces several challenges in its mental healthcare scheme. The mental health sector remains underfunded, with limited resources allocated to mental health care [120]. There is also a critical shortage of trained mental health personnel, which limits the capacity of the healthcare system to meet the demand for services [120]. Stigma and traditional beliefs continue to pose significant barriers to accessing care, with many individuals relying on traditional healers instead of seeking biomedical treatment [115, 121].

### Future directions

**Fig. 2:**
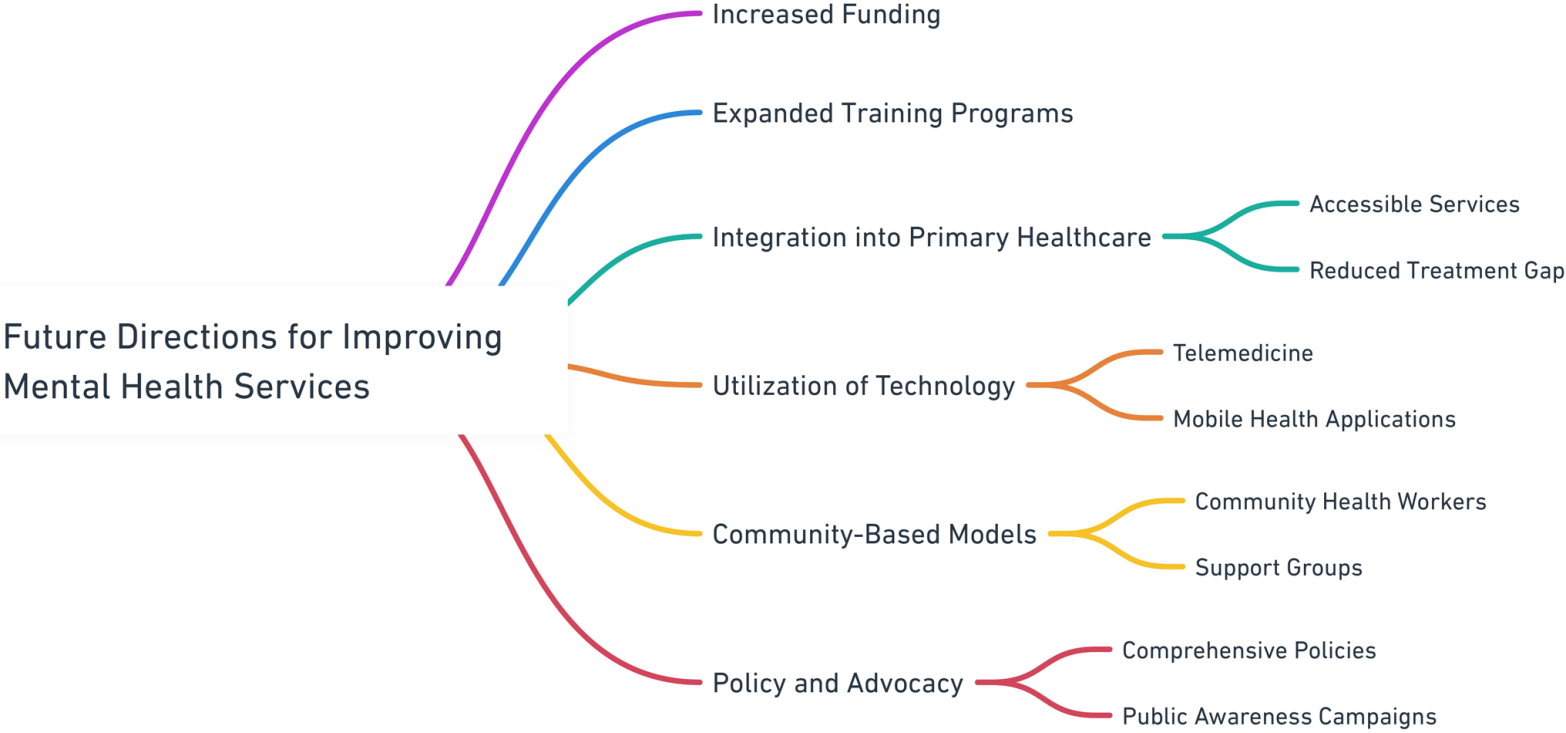
Future Directions for Improving Mental Health Services

Improving mental health rehabilitation services in SSA requires a multifaceted approach that holistically addresses the current challenges and leverages innovative solutions (Fig. 2). At the forefront, it is essential to increase the funding. Adequate financial resources are necessary to build and maintain infrastructure, procure essential medications, and support comprehensive training programs for mental health professionals. These training programs must be expanded and enhanced to ensure that there are a sufficient number of skilled psychiatrists, clinical psychologists, psychiatric nurses, and social care workers. The incorporation of mental health services into primary health care systems is equally important. By integrating mental health care within primary health settings, services become more accessible, particularly in rural and underserved areas, thus reducing the treatment gap. Technology and telemedicine present promising avenues for overcoming geographical barriers and shortages of mental health professionals. Telepsychiatry can provide remote consultation and continuous care, ensuring that even those in the most isolated regions receive support. Mobile health applications can offer mental health support, information, and self-help resources, making mental healthcare more immediate and personalized [122]. Furthermore, digital platforms can serve as invaluable tools for the training and ongoing education of mental health professionals, ensuring that they maintain their current best practices and new developments in the field [83].

Community-based models also play a pivotal role in delivering sustainable and culturally appropriate mental healthcare. Training and deployment of community health workers can bridge the gap between formal healthcare services and the community by providing essential support, education, and referral services. Establishing support forums for individuals with mental health conditions and their families can foster peer support, reduce isolation and promote social reintegration. Collaboration with traditional healers, who are often the first point of contact for mental health issues, can enhance early identification and referral to formal health services, thereby creating a more inclusive and comprehensive care network.

Policy and advocacy efforts are crucial for driving systemic change and ensuring the sustainability of mental health improvements. Governments must develop and implement comprehensive mental health policies that include clear provisions for funding, service delivery, and protection of patient rights. Such policies provide a structured framework for consistent and equitable service provision. In this regard, advocacy plays a pivotal role. Engaging stakeholders, including governments, non-governmental organizations (NGOs), and community leaders, can amplify the call for better mental health services and policies. Public awareness campaigns are essential for educating the public, reducing stigmatization, and encouraging judicious use of mental health services. These campaigns should highlight the importance of mental health care and availability of services, making mental health a community priority [40, 103].

### Limitations

This study offers valuable insights into the mental health landscape and rehabilitation services in SSA; however, several limitations must be acknowledged. First, the scope of the study was restricted by the lack of standardized selection criteria for the countries included in the analysis. This limitation may hinder the generalizability of the findings and reduce the applicability of the conclusions across the region. Ethiopia, Nigeria, Rwanda, South Africa, Tanzania, Kenya, Uganda, and Ghana were chosen to integrate mental health services, community-based programs, and national strategies. Another key limitation is the variability and inconsistency of data sources. Prevalence rates for mental health disorders such as depression, anxiety, PTSD, and schizophrenia vary widely across SSA due to differences in study methodologies, sample sizes, and reporting standards. This inconsistency complicates direct comparisons between countries and may affect the reliability of the aggregated statistics. Furthermore, this study primarily focuses on mental health services, but does not address the broader structural challenges that influence mental health outcomes in SSA. Finally, while this study proposes directions for improving mental health services, it does not provide a comprehensive framework for implementing these recommendations. Future studies could benefit from a focus on scalable evidence-based strategies tailored to the unique sociocultural and economic contexts of SSA. Addressing these limitations through more robust data collection, standardized methodologies, and in-depth analyses of systemic challenges is critical for advancing mental health care in SSA.

## Conclusion

Mental health services in SSA face significant obstacles, including underfunding, shortage of trained professionals, pervasive stigma, inadequate infrastructure, and lack of policies. However, substantial opportunities exist to address these issues. Increasing funding is crucial for building infrastructure, procuring essential medications, supporting comprehensive training programs for mental health practitioners, and integrating mental healthcare services into primary health systems, which will enhance accessibility, particularly in rural areas. Utilizing technology and telemedicine can overcome geographical barriers and professional shortages by offering remote consultation and digital training platforms. Community-based models are essential to provide sustainable and culturally appropriate care. The establishment of support groups and the training of community health workers helps bridge the gaps that exist between formal services and the community. Collaboration with traditional healers can enhance early identification and referral. Governments need to develop robust and effective mental health policies with adequate funding provisions, whereas advocacy campaigns should engage stakeholders in driving systemic changes and reducing stigma. Addressing these challenges through increased funding, innovative solutions, community involvement, and effective policies will greatly improve mental health care services in sub-Saharan Africa, enhancing the well-being of its people.

## Data Availability

No datasets were generated or analysed during the current study.
